# Circular to the Dental Profession

**Published:** 1852-10

**Authors:** 


### CIRCULAR TO THE DENTAL PROFESSION.

The subscriber having at length perfected his new method of mounting mineral teeth, now offers it with increased confidence to the dental profession, from the fact that more than eighty dentists are now using it with entire satisfaction to themselves and patients. That it is all that this circular claims, is the concurrent testimony of all who have tried it, many of whom have successfully tested it by numerous cases worn from twelve to twenty months.

Dr. A. will, in person or by competent agents, visit the principal cities during the present year, and will substantiate, by documentary evidence and actual demonstration, the following statements and facts:

1st. That teeth can be cemented to a plate without platina linings or pivots, so that the strongest man, even with a pair of forceps to hold the plate, cannot remove them with his fingers.

2nd. That this cement is as strong as the teeth themselves.

3rd. That it perfectly fuses to the teeth and plate, forming one solid block or continuous gum, without crack or crevice.

4th. That this gum cannot be acted upon by even the most powerful acids, and has a perfectly natural and life-like appearance.

5th. That it is not liable to scale or crack off. That it may be used for single teeth, as well as full sets, and can be easily transferred to gold plates.

6th. That a temporary set of teeth can be fitted, in a few hours after the teeth are out.

7th. That as the gum absorbs, this same set of teeth, with little labor, can be made to fit the gum again. This may be repeated several times if desired during the process of absorption.

8th. That an entire set of teeth can be put up with one-half the labor, and two-thirds the expense of the usual methods.

9th. That two days' instruction will enable any competent dentist to use this system.

10th. That the teeth of all the manufacturers can be used. Teeth of any form, even pivot teeth, and teeth that would be too short in the old style, will answer in this, since it is not requisite that they should fit the plate, and but little grinding is, therefore, necessary.

11th. If the piece is broken by accident, it can be repaired with less labor than any other style of work.

12th. That this system entirely prevents all warping or springing of plates, even though they may be heated up a dozen times.

13th. That Dr. Allen has been experimenting over eight years, and expended a large amount of money and time, and is fully entitled to the claim of priority, and

That his Patent covers the cementing of teeth together and to the plate by a silicious compound, consequently any formula, if formed of silicious substances, will be an infringement. The Patent Law declares, that an unsuccessful experiment abandoned, will not act as a bar to him who subsequently effects a practical result, even though the same principles may be adopted by both. That repeated attempts have been made within the last fifty years to affect this object is well known. That they have been failures is well understood. The legal rights of Dr. Allen being clearly defined, will be strictly enforced.

In order to bring this improvement at once within the reach of every member of the profession, and to avoid all inducements to evade the patent, the price has been placed as low as any one could expect instruction alone, and much less than it would cost to defend a suit at law. From $150 to $300, according to location, amount of practice, &c., will be charged for an office right.

Those who prefer it, can pay fifty dollars down and a small sum annually during the continuance of the patent. Dentists not residing in the principal cities, by communicating with the undersigned will be informed, at what time and place they can be instructed.

When classes of eight or ten are formed they will receive early attention, and be furnished with everything necessary to prosecute this style of work successfully.

Address, DR. JOHN ALLEN, *Cincinnati, Ohio.*
DR. EDWARD TAYLOR, *Cincinnati.*
DR. B. F. SMITH, *New Orleans.*

### PRIORITY OF INVENTION.

The subscriber is fully aware that such is the nature and importance of his improvement, that it will meet with public favor, yet must pass the same ordeal, and share the same fair and unfair criticisms, that other inventions are subject to.

In all branches of mechanism, as well as in the arts and sciences, as soon as a new principle possessing merit is once discovered and fully developed by one person, there are those who are ever ready to proclaim it theirs. The same indications have also been manifested with reference to this improvement, which has passed through the severest ordeal of any other pertaining to the dental profession for many years—in the Patent Office, in public associations, in private practice, in the face of all opposition, arising from private pique, clashing of interest, professional jealousies, envious competitors, &c., &c., it has withstood all, and now stands upon a much firmer basis than it did one year ago, for it embodies principles which cannot be annihilated by denunciation. It has within the past year been brought foward not only by the inventor, but also by different members of the profession, who have adopted it in their practice to a degree of perfection surpassing the most sanguine expectations, which may be regarded as a very clear indication of what may be looked for in the future.

In order that the dental profession may not be misled with reference to this improvement, by the efforts which have been made to wrest from him his invention, and ascribe to others the credit of having originated and developed it, he deems it proper to notice the main points upon which his calumniators have predicated their grounds of opposition, and based their hopes of success:

The first point that was attempted to be established, was, that he was not the author of his invention, and that he was indebted to a Mr. Steemer for it. This he refuted by ten credible witnesses, several of whose affidavits have been published. In addition to this controverting testimony, Mr. Steemer finally makes the following confession, and calls witnesses to his acknowledgment, which reads thus:

"Cincinnati, March 11th, 1852.

"In justice to Dr. Allen, I hereby certify that I am not the inventor of his new method of setting teeth with continuous gums, and that the first specimens I ever saw of this style of work was at Dr. Allen's office, and this was before I ever attempted anything of the kind myself. And although Dr. Allen did, at one time, try a compound I made for him, I am satisfied he does not use it in his practice.

"I would further state, that the injustice which has been done Dr. Allen through the public press, by misrepresentations, was by the ill advice of others, and I regret exceedingly the injury he has sustained in consequence of my wrong step in this matter, and I take this method of rendering to Dr. Allen that justice and acknowledgment which is his due.

"CHARLES STEEMER.

"Signed in presence of
"C. BURCKHARDT,
"LOIS DOER."

Another attempt was made to cry it down for want of strength. As controverting testimony upon this point, we have the report of the Mississippi Valley Association of Dental Surgery, in which they state they have tried the strength, and believe that no ordinary force, such as is used in mastication, will break the teeth from the plate.

We have also the report of the Ohio College of Dental Surgery, from which we clip the following extract:

"To give an idea of the unyielding nature of Dr. Allen's cement, we will relate one of the many test experiments made in our presence, in the college laboratory, by Dr. Allen:

"He took a slip of platina plate, of the usual thickness, and just wide enough to give bearing on its end to two common sized incisor teeth, and about two inches in length. One end of this was turned up, so as to represent the rim of a plate for the teeth. He then set on this, two incisor teeth, made (expressly for the purpose of testing the cement) without platina pins, backings, holes, or rough surfaces for the cement to cling to, and surrounded them with his composition, which was then fused in a muffle.

"When it was cooled it was passed round, with the request that we would pull off the teeth with our fingers. It was first tried by holding the platina strip in one hand and drawing at the teeth with the other, but no one of us was able to affect them in the least in this way.

"It was then passed round, with a pair of strong pliers to hold the plate, with the request that we would break them off if possible, with that advantage. It passed round uninjured by this test.

"It was next passed round with the pliers as before, with the addition of a piece of paper folded over the teeth, so that we might exert our utmost strength on them without hurting the fingers, when it resisted the trial of nearly all present, but at length one of the teeth was broken in two by the force applied, *leaving the cement, and that part of the tooth embraced by it, still undisturbed on the plate.*

"In conclusion, we would state that we are fully convinced that Dr. Allen is the sole inventor or discoverer of his method of forming artificial gums, and that it is not only practicable, but highly ornamental and useful in the mouth, and that it can only be excelled in strength and durability by the best of natural teeth. As to its capability of resisting the actions of the powerful organs of mastication, (from what we have seen,) we do not believe that they would be broken by any effort of the jaws *short of cracking hickory nuts in the mouth, or of biting in two tenpenny nails.*

Signed :

"Thos. Wood, M. D., *Prof. of Anatomy, &c.*
G. S. Van Emon, *Demonstrator.*
Geo. Mendenhall, M. D., *Prof. of Pathology and Therapeutics.*
Jas. Taylor, M. D., D. D. S., *Professor of Principles and Practice of Dental Surgery.*
Nimrod Hull, Bainbridge, Ohio.
Isaac A. Herring, Kosciusko, Miss.
Y. K. Brewster, Belbrook, Ohio.
M. N. Manlove, Lafayette, Ind.
"W. C. Duncan, Cincinnati, Ohio.
N. P. Allen, Bowling Green, Ky.
J. H. Olds, Circleville, Ohio.
G. L. Paine, Xenia, "
J. C. Whinery, Salem, "
W. S. Jones, Jr., Russellville, Alabama.
John H. Williams, Pittsburgh, Pa.
James T. Irwin, Cincinnati, Ohio.
A. L. Duyers, Greenfield.

"P. S.—The above communication was written and signed without Dr. Allen's solicitation, as a voluntary tribute from his colleagues and students.

"T. WOOD."

In addition to this we have the testimony of some eighty dentists, who have adopted it in their practice, and hundreds of persons who are now wearing this style of work.

Again, it has been stated by his opponents that he is indebted to M. Delabarre for his information upon this subject. This is not so. If M. Delabarre had developed and published a *practical* principle touching this matter, why has it never been carried out before? It would have saved the subscriber from much odium that has been attempted to be cast upon him, for having brought to light that which, until now, has remained obscure.

It has also been stated that Levett is the author of it; this is also controverted by numerous witnesses who have seen specimens of his work before Levett's *enamel* was announced. Levett's object was to cover the *plate* with a very thin coat of varnish or enamel, which flowed at a very low heat by the aid of a common lamp and blow-pipe. The object of the subscriber was to form an artificial gum, strong and solid as the teeth, with strong cohesive properties with which to unite the teeth and plate firmly to each other, which requires a white heat in a furnace to fuse it, specimens of which he showed to Levett's agent, when he first called at the subscriber's office.

He tried some of Levett's preparation for coating plates, by way of experiment, but never adopted its use, nor made it a feature in his practice, as has been asserted by those who would give any one the credit of his invention rather than him. Failing to establish either of the foregoing positions, another effort was made to show that Dr. Hunter was the inventor. This claim was predicated upon the ground that he had (subsequently) made an alleged improvement in block-work, which consisted in uniting single teeth to each other, thus forming a block, which after being fused and cooled, was then soldered on the plate, leaving the teeth and gum disconnected with the plate, which not only admits of more or less secretions, but fails to impart that additional strength and cleanliness to the denture, which a perfect union of the teeth, plate, and artificial gum secures. It is, therefore, upon this single feature, that the whole of the subscriber's invention has been claimed. But this claim is set aside, first, on the ground of priority, as shown by numerous witnesses, and second, by Dr. Hunter's disclaimer, which may be seen on page sixty-six in the Sixth Vol. of the Recorder, which reads thus: "*I would simply state that I do not claim the improvement that consists in sticking teeth to the plate.*" Here he disclaims one very important feature in this improvement, which consists in uniting the teeth and gum firmly to the plate.

Again he says, on page sixty-four, that platina only can be used as a base with certainty, other metals being liable to warp, and this metal being of a soft nature, no great strength can be looked for in it. Here he denounces another important feature in this method of mounting teeth. Platina is preferable to any other metal as a base for this style of work, even where other metals are to be used. It is better to mount the teeth first upon a thin platina base, and then transfer them upon gold, or other plate, as may be desired. Again he says—a fracture, on occurring, what are the means of repair that will prevent a recurrence of the evil? Here he betrays ignorance, as to this method, for a fracture is very easily repaired, but at that time he did not know how it was to be done. But now, after having sent to Washington, and obtained the specifications, he claims it as his own, and says he will use it, and encourages others to do so likewise. He is not the first who has bid defiance to the laws, nor will he be any better able to avoid the consequences by disregarding them, than others who have attempted to set them at defiance. There are some who honestly suppose, that inasmuch as they, or somebody else, had seen the necessity for a certain improvement, and had made experiments with a view to accomplish it, although never carried out by them, and brought into public use, will serve as a bar to a patentee who *has*, from sustaining damages for infringements. This is an erroneous opinion. The law does not recognize the claims of experimenters who have failed to bring out practical improvements. On the contrary, such persons are barred on the ground of abandonment, after two years from the time of their experiments, from setting up subsequent claims that can effect a patentee who has developed and brought into public use that which may have been tried by them, but not rendered practical.

In view of the above considerations, together with the efforts which have been made to wrest from him his invention for mounting artificial teeth upon metallic plates with continuous gums, he forewarns all persons from trafficking, or in any way infringing upon his rights as patentee for the same, as he will most positively protect his patent.

J. ALLEN.

*Extracts from the Proceedings of the Mississppi Valley Association of Dental Surgeons.*

At the recent annual meeting of the Mississippi Valley Association of Dental Surgeons, held in the city of Louisville, Ky., it was resolved that—

"*Whereas*, Dr. J. Allen, of Cincinnati, has been for several years engaged in prosecuting a series of experiments, of which we have been *cognizant*, for the purpose of acquiring principles, by means of which an artificial gum could be formed upon mineral teeth and metallic plates, in such a manner as to unite them firmly to each other, and therefore render more perfect the present method of setting artificial teeth on plate ; and whereas, the results of his experiments have been highly satisfactory to the members of this Association, as exemplified in the specimens he has exhibited ; and believing it due to any member of our society who devotes his time, money, and talents to the advancement of any particular branch of the profession, so that benefit may result therefrom, that some action or commendation from us is necessary :

"Therefore, in view of the great benefit which must result to the profession, and the public generally, from the indefatigable exertions of our brother, Dr. J. Allen, in producing a mineral substance, by the use of which artificial teeth may be more perfectly placed in the mouth, and made to resemble the natural organs of mastication ;

"*Resolved*, That a committee of three be appointed te examine the specimens presented by Dr. Allen, and report to this meeting."

REPORT.

"The committee to whom was referred the preamble and resolution, with the specimens presented by Dr. Allen, would offer the following report, viz.

"That they have examined the teeth cemented together, and to the plate, by Dr. Allen, and have subjected them to the following test :

"That they have tried the strength of adhesion, and believe that no ordinary force, such as used in masticating food, will loosen them from the plate ; indeed, so far as they can judge, the adhesion to the plate is much the same as that effected by solder. Hence, the entire base being secured by the cement, there is greater solidity, and no room for the lodgement of particles of food about the teeth, thus forming substitutes for the block-work, possessing all the advantages of block-work, with more strength, and greater security to the plate.

"They have subjected the gum to the action of nitric and sulphuric acids, and after the pieces had lain overnight in the acids, they find no appreciable effect made on them, although the acids were in a concentrated form.

"The committee are satisfied that this mode of securing the teeth to the plate, recommended by Dr. Allen, possesses cleanliness, strength, and, as far as we can judge, durability. The committee would remark, that in using this cement, the plate used by Dr. Allen is platinum, and pure gold, alloyed 4.100 of platinum; and this greater purity of the metal, which more effectually resists the action of the secretions of the mouth, they regard as advantageous, because it secures the public against the use of inferior gold in mechanical dentistry.

"In view of the labor and expense to which we are satisfied Dr. Allen has been subjected, in bringing this improvement to its present state of perfection, and the advantage to the profession we think its adoption will insure, we therefore recommend the following resolution:

"***Be it Resolved,*** That Dr. Allen deserves all commendation for his indefatigable exertions in the developing and making available a new and important improvement in mechanical dentistry, and that we recommend this improvement to the profession as worthy of their attention.

"JAMES TAYLOR,
"W. H. GODDARD."

### Principles upon which this System is Based.

"With reference to recipes for enamels, I know of none that possess the requisite properties for uniting mineral teeth and metallic plates to each other, in such a manner as to form continuous gums, upon artificial dentures, without either cracking, scaling, or warping the plates upon which they are flowed, unless both sides are covered, which is found to be inadmissible for dental purposes. There are important principles, which must be observed, in order to form a continuous gum without encountering the difficulties aboved named, as the various efforts which have been made to attain this end, and have always proved abortive, clearly show. In the first place, there should be a strong chemical affinity between the component parts of the compound, the teeth, and the plate upon which it is to be fused, in order that a perfect union of the whole should be formed. Among the various metals with which we are most familiar, we find that the linear dilatation of platinum by heat, when raised to 212°, according to Borda, is one part in 1,167, which is the least of any of the metals. Palladium, according to Wollaston, 1 in 1,000; Antimony, 1 in 1,123; Gold, 1 in 713; Copper, 1 in 584; Brass, 1 in 548; Silver, 1 in 505; Tin, 1 in 438; Lead, 1 in 345; Zinc, 1 in 339.

"Among the minerals we find that the same degree of temperature will produce a linear expansion. Marble, from St. Beat, 1 in 2,391; Stone, from St. Pernon, 1 in 2,304; Stone from St. Leu, 1 in 1,541. The clay which is obtained in Dorsetshire and Devonshire, 1 in 2,123. In Wedgewood's wares, a large proportion of this material is used. Cornish stone, 1 in 1,521; English flint glass, 1 in 1,248; French plate, 1 in 1,147; Sulphate of Barytes, 1 in

1,204; Sulphate of Strontites, 1 in 1,128; Asbestos, 1 in 1,769; pure Silex, 1 in 1,662.

"From the metal above named, I select platina as the most suitable for plates for my new style of work, because the linear dilatation and contraction of this metal when exposed to extreme temperature of heat and cold, are less than in any other metal. The mineral compound to be employed in forming a continuous gum upon artificial dentures, should possess the same degree of resisting power as the plate upon which it is to be fused, otherwise the contractility of the mineral in cooling after fusion, (if greater than that of the plate,) would either cause a cracking of the cement, or warping of the plate, which in either case would be highly detrimental. The same result would follow if the linear expansion or contraction of the metal plate was greater or less than that of the mineral. Hence the importance of having the same degree of dilatation and contractility in the metal and the mineral. Therefore, if substances be used as fluxes, etc., for uniting the minerals which contract much, other substances more refractory in their nature should be added to the compound, such for instance as Strontia, Devonshire clay, or Wedgewood, Asbestos or stone from St. Pernon or St. Leu. These substances duly proportioned and properly prepared may be employed to great advantage in my silicious compound, which I employ in the construction of continuous artificial gums, as exemplified in my new style of work."

The following are some of the persons now using this style of work, and we advise those who wish to remove the last vestige of a doubt, to write to some one of the number in whom they may have confidence, at once:

Dr. Guild, Boston.
Drs. Bridges & Smith, Brooklyn, New York.
Dr. Smith, Terre-Haute, Ind.
Dr. Goddard, Louisville, Ky.
Dr. Griffith.
Dr. Wright, Pittsburgh, Pa.
Dr. Herring, Kosciusko, Miss.
Dr. White, Utica, N. Y.
Dr. Barlow, New York.
Dr. Hawes, New York.
Dr. Strickland, Cleveland, Ohio.
Drs. J. & E. Taylor, Cincinnati, O.
Dr. Hawes & Bro., Providence, R. I.
Dr. Allen, Editor Dental Recorder.
C. C. Porter, Block-workman.

